# The Family Caregiving Environment Associates with Adolescent Patients’ Severity of Eating Disorder and Interpersonal Problems: A Cross-Sectional Study

**DOI:** 10.3390/children10020237

**Published:** 2023-01-28

**Authors:** Giorgia Baradel, Livio Provenzi, Matteo Chiappedi, Marika Orlandi, Arianna Vecchio, Renato Borgatti, Martina Maria Mensi

**Affiliations:** 1 Department of Brain and Behavioural Sciences, University of Pavia, 27100 Pavia, Italy; 2 Child Neurology and Psychiatry Unit, IRCCS Mondino Foundation, 27100 Pavia, Italy; 3 Vigevano Child Neurology and Psychiatry Unit, ASST Pavia, 27100 Pavia, Italy

**Keywords:** adolescents, anorexia nervosa, family-centred approach, family functioning, interactive behaviours, Lausanne Trilogue Play, mental disorders, restrictive eating disorders

## Abstract

The quality of family interactions may be a critical factor for restrictive eating disorders (REDs). Adolescent patients with RED have interpersonal problems that can be inferred by observing their behaviours during family interactions. To date, the assessment of the association among RED severity, interpersonal problems, and patients’ interactive behaviours in the family is partially explored. This cross-sectional study aimed to explore how adolescent patients’ interactive behaviours observed during the Lausanne Trilogue Play—clinical version (LTPc) were associated with both RED severity and interpersonal problems. Sixty adolescent patients completed the EDI-3 questionnaire to assess RED severity using the Eating Disorder Risk Composite (EDRC) and Interpersonal Problems Composite (IPC) subscales. Moreover, patients and their parents took part in the LTPc, and patients’ interactive behaviours were coded as participation, organization, focal attention, and affective contact in all the LTPc four phases. A significant association emerged between patients’ interactive behaviours during the LTPc triadic phase and both EDRC and IPC. Better patients’ organization and affective contact significantly correlated with lower RED severity and fewer interpersonal problems. These findings suggest that investigating the quality of family relationships and patients’ interactive behaviours may contribute to better identifying adolescent patients at risk for more severe conditions.

## 1. Introduction

Restrictive eating disorders (REDs) are a heterogeneous group of serious psychiatric diseases characterised by restricted oral intake [1] and associated with an increased incidence among young people in recent years, especially in the high-risk group of 15- to 19-year-old girls [2,3]. Recent studies have been directed towards a better understanding of the aetiology and mechanisms governing eating disorders.

The onset of REDs is influenced by several individual factors concerning body imaging and psychological aspects, which work together for the maintenance, evolution, and severity of the disease [4,5]. Moreover, studies on interactive patterns have been described in REDs adolescents’ families. They show new considerations to better understand aetiological aspects and to hypothesise new perspectives on treatment and prevention of REDs. In connection with this aspect, the presence of interpersonal problems has been described in patients affected by REDs not only in a social context but also in the family context, typically in families with collusive and dysfunctional functioning [6,7,8,9]. Interpersonal problems include a wide range of difficulties related to social functioning and the process by which interactions with others are internalized [10]. According to recent studies, such difficulties are related to avoiding expressing feelings to others, prioritizing others’ feelings over one’s own, and high levels of social anxiety and social comparison [11]. In the recent literature, interpersonal problems have also been defined as a “core” component of REDs and as a risk factor for the development and maintenance of the disorder [12]. Scientific evidence suggests that family functioning plays an important role in the establishment and evolution of the pathology [13,14], and a therapeutic work on it has strong short- and long-term beneficial outcomes [15,16]. On this subject, interpersonal problems can also be considered for the treatment of the disorder and reducing AN symptoms [1], particularly within a dysfunctional family context [6,7]. Improvements in the family’s communication, behaviours and the ability to internalize emotions have been associated with a period of REDs patient hospitalization [17], proving the correlation between this eating disorder and family interactions. Residential treatment has also been correlated to changes in being interpersonally distant and overly interpersonally controlling [18]. Considering these data, since the presence of interpersonal problems is related to family functioning, a specific work on it could contribute to the improvement of the interpersonal problems and thus of the disease.

Recent literature on family functioning is often based on subjective data collected from self-reported questionnaire by families and patient. This work allows to focus on a recognised point of view about family dynamics, studying family from different sub dimensions. The Lausanne Trilogue Play (LTP) [14] has been widely used to assess family and patients’ interactive behaviours. During this validated observational instrument, the patient interacts with both his/her parents to organize an activity together in four phases (Table 1). Through a coding scheme drawn up by Malagoli, Togliatti, and Mazzoni [19], it is possible to score the patients’ interactive behaviour for several parameters (Table 2). Studying families of patients with RED, all specific functional levels of patients’ interactive behaviour are found to be less adequate than in families without a patient with RED. In detail, levels of organization (OR) and affective contact (AC) seem to be of particular concern in patients with RED [20] since it is evident that the patient tries to manage the relationship between the parents even when not required by relieving the conflictual tension, preferring a dyadic relationship to a triadic one [6,20]. Literature states that patients with REDs reveal significantly higher difficulties to feel and regulate affect [21], which is also correlated with the severity of autonomic dysregulation [22]. Moreover, in the third phase of the play, a general difficulty in interaction is observed in the triad that also involves the mentioned emotional aspects [6,23].

To date, the assessment of the relationships between REDs severity, interpersonal problems, and patients’ interactive behaviours in the family has been only partially investigated. As such, the present study aims to bridge the gap in the literature with a three-fold aim: (a) to explore the relationship between the patients’ interactive behaviours across the LTPc phases and RED severity, (b) to explore the relationship between the patients’ interactive behaviours across the LTPc phases and interpersonal problems, and (c) to assess the association of RED and interpersonal problems focusing on specific functioning levels (e.g., PA, OR, FA, and AC). 

Relying on what is known in the literature, we aim to provide wider information about the connection between REDs characteristics and how the patient acts in the family environment. We hypothesized that, in adolescents with REDs, better patient interactive behaviours (observed during dialogue with the parents) are associated with improved interpersonal problems and lower disease severity (a–b). We also hypothesised that LTPc functioning levels, which require more relational exchange, reflect the disease’s developments.

The originality of this work consists in using the LTPc as an objective instrument to assess family functioning and interweaving it with a subjective assessment constituted by the EDI-3 questionnaire. To our knowledge, this is the first work that relates the family aspects observed with this methodology and the psychological aspects relating to the severity of the pathology. A linear association between these variables could support the idea of a family treatment to work on the patient’s difficulty and to modify not only the family functioning but also the development and maintenance of the disease. 

The description of each section of the paper is available in detail in the record checklist attached to the supplementary material.

## 2. Materials and Methods

### 2.1. Participants

**Figure 1:**
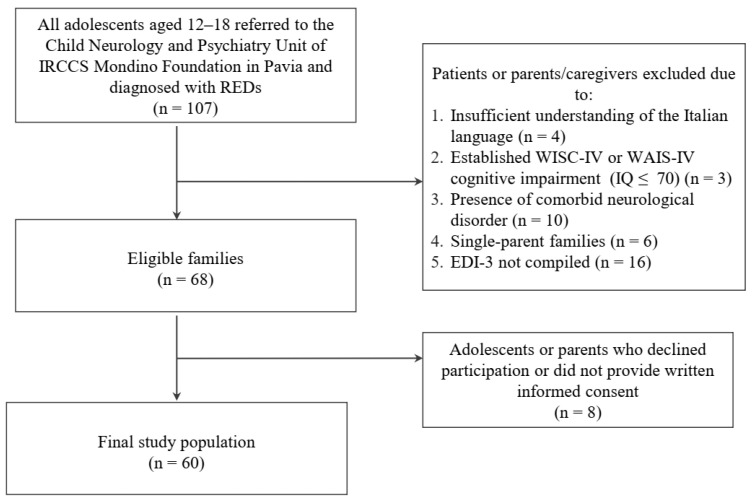
Study population flowchart.

Sixty adolescent patients were enrolled at the Child Neurology and Psychiatry Unit of the tertiary care Scientific Hospitalization and Treatment Institution Mondino Foundation in Pavia, a city in northern Italy. Inclusion criteria were being between the ages of 11 and 18 (extremes included) and having received a diagnosis of RED according to the Diagnostic and Statistical Manual of Mental Disorders (DSM-5) criteria [24]. RED diagnosis could include any restrictive and binge-eating/purging subtypes of anorexia nervosa (AN), atypical anorexia nervosa (A-AN), or other specified eating disorders with restrictive characteristics. Patients with any comorbid neurological disorders were not eligible for the study. REDs diagnoses were confirmed by administering the DSM-based Kiddie Schedule for Affective Disorders and Schizophrenia (K-SADS-PL DSM-5) [25,26], which was also used to exclude patients with any comorbid neurological disorders (e.g., autism spectrum disorder, or other psychiatric diseases). Intellectual disability was assessed using age-appropriate Wechsler intelligence scales [27,28]. and personality disorders in structuring were evaluated through the Structured Clinical Interview for DSM-5—Personality Disorders (SCID-5 PD) [29]. We also excluded patients and parents who did not understand the Italian language. Figure 1 shows the study population flowchart. The cross-sectional study was approved by the Ethics Committee of the Policlinico San Matteo in Pavia (Protocol ID: P-20170016006) and performed according to the Reporting of studies Conducted using Observational Routinely collected health Data (RECORD) statement (Supplementary Table S1). All patients and their caregivers provided written informed consent to the study. The authors assert that all procedures contributing to this work comply with the ethical standards of the relevant national and institutional committees on human experimentation and with the Helsinki Declaration of 1964 and its later amendments. The dataset is available upon request in Zenodo [30].

### 2.2. Procedures and Measures

On admission, patients and parents were interviewed about medical and family history, followed by neurologic and psychiatric evaluation and a complete clinical neurologic examination. Later, adolescent patients were asked to complete the Eating Disorder Inventory (EDI-3) questionnaire [31,32]. Each of the 91 items is rated on a 6-point Likert scale (range: 1, never; 6, always) and grouped into 12 subscales: three specific to eating disorders and nine focused on patients’ psychological characteristics. For the aims of the present study, two composite scores were considered. The Eating Disorder Risk Composite (EDRC) included reports of DT (drive for thinness), BD (body dissatisfaction), and B (bulimia). The Interpersonal Problems Composite (IPC) included items referred to as interpersonal alienation and interceptive deficits. This work uses as a sample a patient who was diagnosed almost at the same time as the EDI-3 was delivered: as shown in some studies [5,33,34], in this work, the drive for thinness, body dissatisfaction, and bulimia are not considered in the risk assessment but in the severity of illness. The use of this self-compiled questionnaire is an innovative methodology to focus on the patient’s voice about the disease to understand how the external aspects can modify the pathologic condition.

In addition, the patients’ interactive behaviour in the family was assessed using the LTPc [19]. The LTPc was videotaped and subsequently coded by two trained independent coders. The procedure [6,8,20] is structured in four phases and requires family members to complete a specific interactive task (Table 1). 

For the aims of the present study and consistent with previous research [6], we considered the third phase in which both parents actively interact with the patient. The coding system of LTPc includes four patient functioning levels: participation (PA), organization (OR), focal attention (FA), and affective contact (AC) (Table 2). For each functioning level, the patients receive a score of 0 (dysfunctional), 1 (partially functional), or 2 (functional). Patients’ interactive behaviour in the family was finally quantified using a global patient’s functioning score (range: 0 to 8) obtained for each LTPc phase by summing the scores of the specific functioning levels.

### 2.3. Plan of Statistical Analyses

The analyses were conducted using IBM SPSS 27 [35] and R [36] for Windows, setting *p* < 0.05. Stepwise linear regression models were used to test linear and quadratic associations of patient scores during LTPc phases and both EDRC and IPC scores. Separate analyses of variance (ANOVAs) were used to assess the difference in EDRC and IPC scores among patients with high (2), moderate (1), and low (0) scores in the different levels of patient functioning during phase 3 (triadic phase).

## 3. Results

Table 3 shows descriptive statistics and clinical characteristics of the sample.

**Figure 2:**
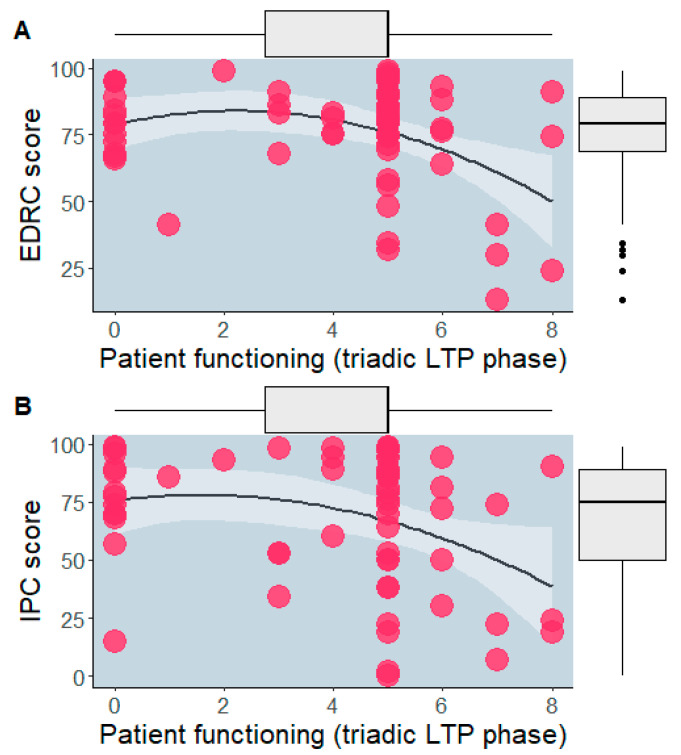
Associations between triadic phase score and EDRC (**A**) and IPC (**B**) ones.

Significant quadratic associations emerged for the patient’s score during the triadic phase with EDRC score (*R*^2^ = 0.11, F = 7.37, *p* = 0.009, *B* = −0.40, t = −2.72, *p* = 0.009, 95% CI [−0.69:−0.10] (excluded linear association, *p* = 0.499)) and IPC score (*R*^2^ = 0.10, F = 6.37, *p* = 0.014, *B* = −0.54, t = −2.52, *p* = 0.014, 95% CI [−0.97:−0.11] (excluded linear association, *p* = 0.142)) (Figure 2). No significant linear or quadratic associations emerged for the other LTPc phases.

**Figure 3:**
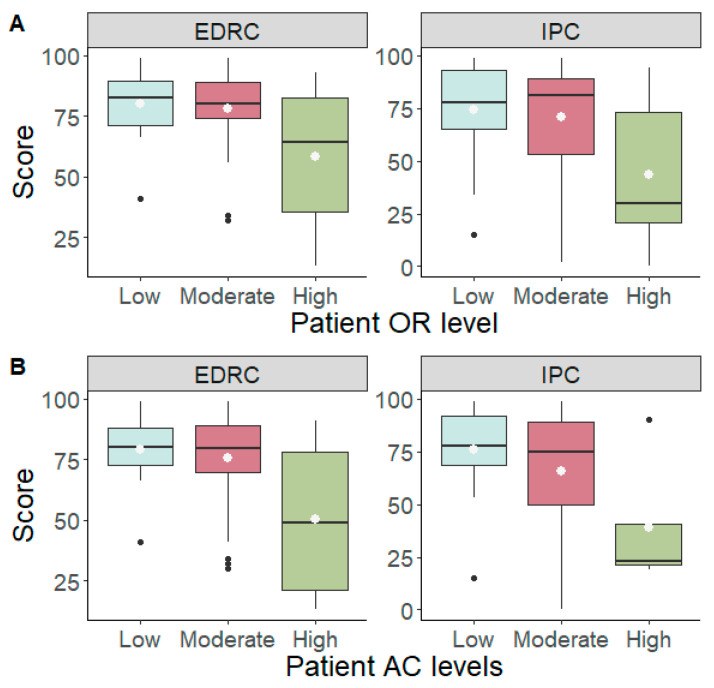
Significances between OR (**A**) and AC (**B**) and both EDRC and IPC.

Significant differences emerged for the patient’s OR during the triadic phase in EDRC score (*F* (2.57) = 5.51, *p* = 0.007, *η*^2^*p* = 0.16) and IPC score (*F* (2.57) = 5.19, *p* = 0.009, *η*^2^*p* = 0.15). Post hoc analyses revealed that patients with high OR score had lower EDRC and IPC score compared to counterparts with low and moderate OR score. Additionally, significant differences emerged for the patient’s AC during phase 3 (triadic phase) in EDRC score (*F* (2.57) = 3.82, *p* = 0.028, *η*^2^*p* = 0.12) and IPC score (*F* (2.57) = 3.04, *p* = 0.055, *η*^2^*p* = 0.10). Post hoc analyses revealed that patients with high AC score had lower EDRC and IPC score compared to counterparts with low and moderate AC score (Figure 3).

## 4. Discussion

The primary aim of this study was to assess the presence of a significant association between patients’ interactive behaviours during the triadic interaction phase and both RED’s severity and interpersonal problems. As expressed in the hypothesis, our findings suggest that better interactive behaviours of patients within the family corresponded to lower severity of RED. Since this correlation is found only in the triadic phase, and it is probably related to the other two components of the family, this is in line with recent literature. Results refer to the triadic phase, in which a recent study [6] observed worse family coordination, with marked difficulties in respecting one’s roles, maintaining joint focal attention, and finally, sharing emotions and affection. Family components could also play an important role in the complex pathogenetic, maintenance, and treatment mechanisms of AN [13,37,38]

Furthermore, while some studies have focused on the subjectivity of the patient and have limited themselves to describing dyadic relationships, giving these a particularly important character in the development of the disorder, other studies have reported that severity was correlated with familial expressiveness [39]. In particular, Lyke et al. [37] found a correlation between general family functioning, affective responsiveness, and risk of pathology. The novelty of our study was the use of the LTPc as an objective technique for assessing family functioning as scored by a trained clinician [40,41]. This observation allowed us to assess the triangular coordination in its complexity and not only the patient–parent dyadic mode: this peculiarity allows the clinician to study the real family relationship by simulating a dynamic even closer to daily life.

This study also showed a correlation between the patients’ interactive behaviours observed in the third phase and interpersonal problems (IPC): the better the patient’s interactive behaviour, the lower the IPC. Previous studies attributed the patient’s interpersonal problems to a severe psychopathological disorder of adolescent development [42], while our results suggest that the patient’s interactive difficulties can be explained by the abnormal triadic interaction observed in the third phase of LTPc. The correlation between the patients’ interactive behaviour and IPC was significant only in the third phase: this is in line with the study conducted by Balottin et al. [8], in which difficulties in the family relationship emerged almost exclusively in the triadic level and not the dyadic one. Furthermore, adolescents with more impaired personal functioning seemed to prefer dyadic interactions with one parent at a time even when the mother–father–son/daughter triad was asked to play together (as in phase 3). The patient’s impairment in establishing and maintaining healthy and meaningful relationships with others is associated with an elective difficulty in triadic interpersonal interactions, which means a specific difficulty in overcoming the more infantile developmental stages (dyadic vs. triadic relationship) and achieving a triadic relationship with a rich and flexible ability to interact with others.

In addition, this study explored which different levels of patients’ interactive behaviours could influence the severity of RED and interpersonal problems. It appeared that during the third phase, there were significant correlations between OR and both EDRC and IPC and between AC and both EDRC and IPC. In other words, the higher the OR and AC scores, the lower the EDRC and IPC for both aspects of functioning. Considering the organization, the data are in line with the recent literature that states that eating disorders are linked to a specific difficulty in respecting the role of the family [6]. In a study by Mensi et al. [20], it was seen that mothers appeared very involved even when they were not called to play, while fathers were less involved, almost as a defensive reaction to their son/daughter’s illness. The families had difficulty functioning as “three together”, with a strong tendency towards dyadic functioning with the exclusion of one participant. Unlike recent studies [37] in which the correlation between family roles and the risk of increased severity of disease did not emerge, in the present study, a significant link was observed. One might wonder why the findings differ between different studies. First, this difference could be due to methodological issues. The use of an objective evaluation tool as LTPc allowed an analysis freer from personal judgments and allowed the clinician a more careful observation of family dynamics. This may suggest that the choice in the use of certain evaluation techniques (tests or observational dynamics) is important, as the results can provide different points of view and insights on the pathology: from the data obtained, it is also possible to define the optimal therapeutic process. 

Moreover, it should be noted that in the present study, unlike previously mentioned studies, we enrolled patients under the age of 18. This element allows us to consider the complexity of patients since they would be in the moment of transition of life in which many elements of their personality would still be in structuring. Unlike a more mature sample, the one in this study refers to constantly moving dynamics that inevitably require support and external guidance. In this sense, the detailed and punctual study of the family organization is fundamental to understanding the dynamics that could affect the pathology. As such, in future research, it is important to investigate family functioning and the severity of RED in multiple and diverse subpopulations of patients.

There was also a significant correlation between AC and both EDRC and IPC. This is in line with the findings of other studies conducted on similar samples of female adolescents affected by REDs, particularly AN [37], in which during the third phase of LTP, the affective quality of the interaction was compromised by difficulties of the father and son/daughter in maintaining contact and sharing effects simultaneously, demonstrating that the father’s role is fundamental in the potential emotional deficit of families with a son/daughter with RED [23,41]. On the other hand, the maternal figure is described as hyper-involved in the relationship, altering the balance of the triad [6]. Beyond the paternal role, overall, it is known that parents show a high degree of distension, competitiveness, and conflict, which often makes communication between them impossible without the son/daughter’s intervention: this seems to have a negative impact not only on the partners but also on the family in general and therefore on the patient. It is not only the influence of each parent on the son/daughter separately that provides a matrix for the son/daughter’s development but also, and even more significantly, the affective regulation of the parental couple expressed in the complex relational dynamic [42,43]. This study reinforces the hypothesis that emotional contact is one of the dynamics involved in the development of eating disorders and interpersonal difficulties. 

Nevertheless, our study had limitations. First, the sample size is relatively small, mainly due to the need to include only families available to take part in the LTPc. This limits the generalization of the current results, which need to be replicated in larger samples in the future. Second, we focused on REDs, excluding other eating disorders such as bulimia nervosa and binge-eating disorder. Future research should investigate whether these findings apply to all eating disorder conditions. Finally, a healthy control group was not included in this study. 

We believe that the use of a self-reported questionnaire can lead to considering a different point of view of the disease; it can also provide information to better study the differences between the doctor’s objective and the patient’s subjective perception of the disease. Furthermore, the availability of observational measures of family interaction patterns is a strength of this study: parents’ opinions could be different from the clinical impression, and this aspect could be an interesting starting point for future studies. We think that our results are also useful in clinical practice, as these observations allow us to structure a new diagnostic and therapeutic approach to REDs. The use of a validated system such as LTPc, administered by trained people, can be very useful in monitoring the progress of the disease. Its use could correct possible weaknesses in family functioning, improving interpersonal problems and the history of the disease.

## 5. Conclusions

Families of adolescent patients diagnosed with REDs usually develop specific dysfunctional interactive patterns characterized by a collusive alliance. At the same time, these patients often manifest interpersonal problems. Notably, this study further adds that there may be a significant association between family functioning and the presence of interpersonal problems. These findings have critical implications for clinical practice. Several studies suggest prioritizing a family-centred approach to the assessment and treatment of REDs [37,39,44].

A detailed and punctual study of the patients’ interactive behaviours and, to a large extent, of the family makes possible to identify a worse restrictive eating disorder situation. Therefore, the interaction of the whole family is a clear, specific aspect for the identification of a possible higher-risk situation. The presence of subthreshold symptoms, in association with a low LTPc score of the dysfunctional aspects viewed during LTPc, induces one to think about a higher-risk situation and allows suggesting a potential preventive intervention [37,39,44]. 

## Figures and Tables

**Table 1:** Description of LTPc phases.

Phase	Description
1	Mother/father interacts with son/daughter, while the other parent is simply present.
2	Father/mother interacts with son/daughter, while the other parent is simply present.
3	The parents interact together with their son/daughter.
4	The parents continue to interact with each other without involving the son/daughter.

**Table 2:** LTPc functional levels.

Functional Level	Description	How to Measure
Participation (PA)	Ability to get involved in the same interactive space, getting in touch with the other family members. It indicates the inclusion or the exclusion of a member of the triad.	Note how the participant places his body in the inter-active field, if he/she sits correctly, and if he/she orients his/her body to other family members and the task.
Organization (OR)	The capacity of each participant to play a role coherent with the different parts of the play: the roles played by each parent and the son/daughter have to be different according to the different phases	Note if each participant plays her/his role.
Focal Attention (FA)	Ability to reach and maintain a joint attentive focus shared by the triad during the play.	Note whether each of the participants (regardless of their role in a specific phase) pays attention to the interactive elements, ongoing activities, and actions of the other participants, sharing meanings with each other through glances, gestures, and words.
Affective Contact (AC)	Emotional sharing and the reciprocity and communion of affections.	Note ways of looking, physical contact, verbal communication of affections, and reinforcements within the triad

**Table 3:** Descriptive statistics of the sample.

		Complete Data	
		(*n* = 60)	
		**Mean**	**SD**
Age (months)		180.12	111.480
Weight, kg		40.773	9.8736
Height, cm		158.133	7.1247
BMI kg/m^2^		16.26	3.5995
Weight loss, kg		95.958	274.7674
		**N**	**%**
Sex	Female	59	98%
	Male	1	2%
		**N**	**%**
Patient’s social withdrawal	Yes	5	8%
	No	55	92%
Patient’s school withdrawal	Yes	6	10%
	No	54	90%
Menarche	Yes	46	77%
	No	14	23%
Period	Primary amenorrhea	9	20%
	Secondary amenorrhea	29	63%
	Irregular period	8	17%
Binge eating	Yes	17	28%
	No	43	72%
Dysmorphophobia	Yes	52	87%
	No	8	13%
Obsessive-compulsive behaviours	Yes	25	42%
	No	35	58%
Hyperactivity	Yes	31	52%
	No	29	48%
Elimination conducts	Yes	15	25%
	No	45	75%
Family history of psychiatric disorders	Yes	37	62%
	No	23	38%
Family history of eating disorders	Yes	4	7%
	No	56	93%
Treatment	Day hospital	7	14%
	Diet	21	42%
	Psychiatric	22	44%
		**Mean**	**SD**
Paternal educational level		61.97	216.813
Maternal educational level		62.82	216.607
Family socioeconomic status		103.500	241.6933

## Data Availability

Data are available upon reasonable request from Zenodo.

## Supplementary Materials

The following supporting information can be downloaded at: https://www.mdpi.com/article/10.3390/children10020237/s1, Table S1: The RECORD statement—checklist of items, extended from the STROBE statement, that should be reported in observational studies using routinely collected health data.

## Abbreviations

A-AN	atypical anorexia nervosa
AC	affective contact
AN	anorexia nervosa
B	bulimia
BD	body dissatisfaction
BMI	body mass index
DT	drive for thinness
EDI-3	Eating Disorder Inventory-3
EDRC	Eating Disorder Risk Composite
FA	focal attention
IPC	Interpersonal Problems Composite
K-SADS-PL	Kiddie Schedule for Affective Disorders and Schizophrenia-Present and Lifetime
LTPc	Lausanne Trilogue Play—clinical version
OR	organization
PA	participation
REDs	restrictive eating disorders
SCID-5-PD	Structured Clinical Interview for DSM-5—Personality Disorders
